# Pod pepper vein yellows virus, a new recombinant polerovirus infecting *Capsicum frutescens* in Yunnan province, China

**DOI:** 10.1186/s12985-021-01511-5

**Published:** 2021-02-23

**Authors:** Kuangjie Zhao, Yueyan Yin, Mengying Hua, Shaoxiang Wang, Xiaohan Mo, Enping Yuan, Hongying Zheng, Lin Lin, Hairu Chen, Yuwen Lu, Jianping Chen, Jiejun Peng, Fei Yan

**Affiliations:** 1 State Key Laboratory for Managing Biotic and Chemical Threats To the Quality and Safety of Agroproducts, Institute of Plant Virology, Ningbo University, Ningbo, 315211 Zhejiang China; 2 Institute of Alpine Economic Plants, Yunnan Academy of Agricultural Sciences, Lijiang, 674100 Yunnan China; 3 College of Plant Protection, Yunnan Agricultural University, Kunming, 650201 Yunnan China; 4 Wenshan Academy of Agricultural Sciences, Wenshan, 663000 Yunnan China; 5 Yunnan Academy of Tobacco Agricultural Sciences, Kunming, 650021 Yunnan China

**Keywords:** Pepper vein yellows virus, Recombination, *Polerovirus*, Readthrough domain

## Abstract

Pepper vein yellows viruses (PeVYV) are phloem-restricted viruses in the genus *Polerovirus*, family *Luteoviridae*. Typical viral symptoms of PeVYV including interveinal yellowing of leaves and upward leaf curling were observed in pod pepper plants (*Capsicum frutescens*) growing in Wenshan city, Yunnan province, China. The complete genome sequence of a virus from a sample of these plants was determined by next-generation sequencing and RT-PCR. Pod pepper vein yellows virus (PoPeVYV) (MT188667) has a genome of 6015 nucleotides, and the characteristic genome organization of a member of the genus *Polerovirus*. In the 5′ half of its genome (encoding P0 to P4), PoPeVYV is most similar (93.1% nt identity) to PeVYV-3 (*Pepper vein yellows virus 3*) (KP326573) but diverges greatly in the 3′-part encoding P5, where it is most similar (91.7% nt identity) to tobacco vein distorting virus (TVDV, EF529624) suggesting a recombinant origin. Recombination analysis predicted a single recombination event affecting nucleotide positions 4126 to 5192 nt, with PeVYV-3 as the major parent but with the region 4126–5192 nt derived from TVDV as the minor parent. A full-length clone of PoPeVYV was constructed and shown to be infectious in *C. frutescens* by RT-PCR and the presence of icosahedral viral particles.

## Background

Pepper vein yellows viruses (PeVYV) are phloem-restricted viruses in the genus *Polerovirus*, family *Luteoviridae* and are currently classified into six species (International Committee on Taxonomy of Viruses [ICTV] 2019 release), named *Pepper vein yellows virus 1* to *6* [1–6]. They have 86.2–94.6% nucleotide identity between them. Polerovirus genomes have seven open reading frames (ORF0 to ORF5 and ORF3a), putatively encoding proteins P0 to P5 and P3a [6]. Recombination is an important source of genetic variability in viruses, particularly for viruses possessing an RNA genome. PeVYVs have quite close nucleotide similarities to tobacco vein distorting virus (TVDV) in the 5′ half of their genomes, and are considered to have arisen from recombination between TVDV and other poleroviruses [4, 7, 8]. We here report a new recombinant of PeVYV with high identity to PeVYV-3 in the 5′ half of its genome and to TVDV in the 3′ part.

## Main text

Pod pepper (*Capsicum frutescens*) is widely planted in China, especially around Wenshan city, Yunnan province, and viral diseases have now also become a major threat to pepper production in Yunnan. During July 2019, 89 pepper leaf samples were collected from three different fields in Wenshan. These samples were collected from Da Longshu village (a, 45 samples), Bai Shiyan village (b, 28 samples) and Da Shuduan village (c, 16 samples). All had typical viral symptoms of interveinal leaf yellowing and fruit discoloration (Additional file 2: Fig. S1). These leaves (0.4 g per sample) were mixed into three pooled samples by origin, and then sent to the Central Laboratory of Zhejiang Academy of Agricultural Sciences (Hangzhou, China) for Next-generation RNA-Seq sequencing (NGS). A total amount of 1 μg RNA per sample was used as input material for the RNA sample preparations. RNA integrity was checked by Agilent 2100 Bioanalyzer (Agilent Technologies). The TruSeq RNA Sample Preparation Kit (Illumina) was used to construct cDNA libraries according to the manufacturer’s instructions. An Illumina NovaSeq 6000 platform with PE150 bp and CLC Genomics Workbench 20 (QIAGEN) was used for sequencing and data analysis. A total of 36,430,754 (a: 17,322,690, b: 11,735,694 and c: 7,372,370) paired-end reads were obtained, and 432,848 contigs (a: 143,689, b: 113,012 and c: 176,147) were generated de novo and compared with sequences in the GenBank nt using BLASTn, and 7 contigs were identified with E-values of zero. Contig_62 was 5992 nt long and had high identities (> 87.5%) to the genome of PeVYV-3 (*Pepper vein yellows virus 3*) (KP326573 [3];) and the other contigs were matched with Chilli ringspot virus (ChiRSV, 3 contigs), Chilli veinal mottle virus (ChiVMV, 1 contig), Tomato zonate spot virus (TZSV, 1 contig) and Cucumber mosaic virus (CMV, 1 contig).

In order to verify the virus sequences, total RNA was isolated from each sample using TRIzol™ Reagent (Invitrogen) in compliance with the manufacturer’s instructions. Reverse transcription (RT) polymerase chain reaction (PCR) analyses were performed using the ReverTra AceTM qPCR RT Master Mix (Toyobo) and KOD-plus-Neo (Toyobo) following the manufacturer’s protocol. RT was performed at 42 °C for 60 min with random primers followed by 72 °C for 10 min. The cycling conditions for the subsequent PCR were: 98 °C 3 min, and then 35 cycles of 98 °C for 30 s, 55 °C for 30 s, 68 °C for 1 kb/min; and 68 °C for 10 min. Primers designed from Contig_62 were used to amplify a coat protein (CP) fragment of the virus (PeVYV-CP) (Additional file 1: Table S1). Fragments of the expected size (621 bp) were obtained in 58 of the 89 symptomatic samples but not from healthy plants (raised from seed in the laboratory). After adding dATP at the 3′-terminal of the PCR products using1 μL Taq™ (TaKaRa) at 72 °C for 10 min, they were cloned into pGEM-T Easy vector (Promega) and sequenced commercially (Sangon Biotech). The sequences were aligned using MUSCLE, and pairwise nucleotide sequence comparisons were done using the SDT (Species Demarcation Tool) v1.2 program [9, 10]. The 116 amplicons were 93.0–98.4% identical to PeVYV-3, and so we tentatively designated this virus in pod pepper as Pod Pepper vein yellows virus (PoPeVYV).

**Fig. 1:**
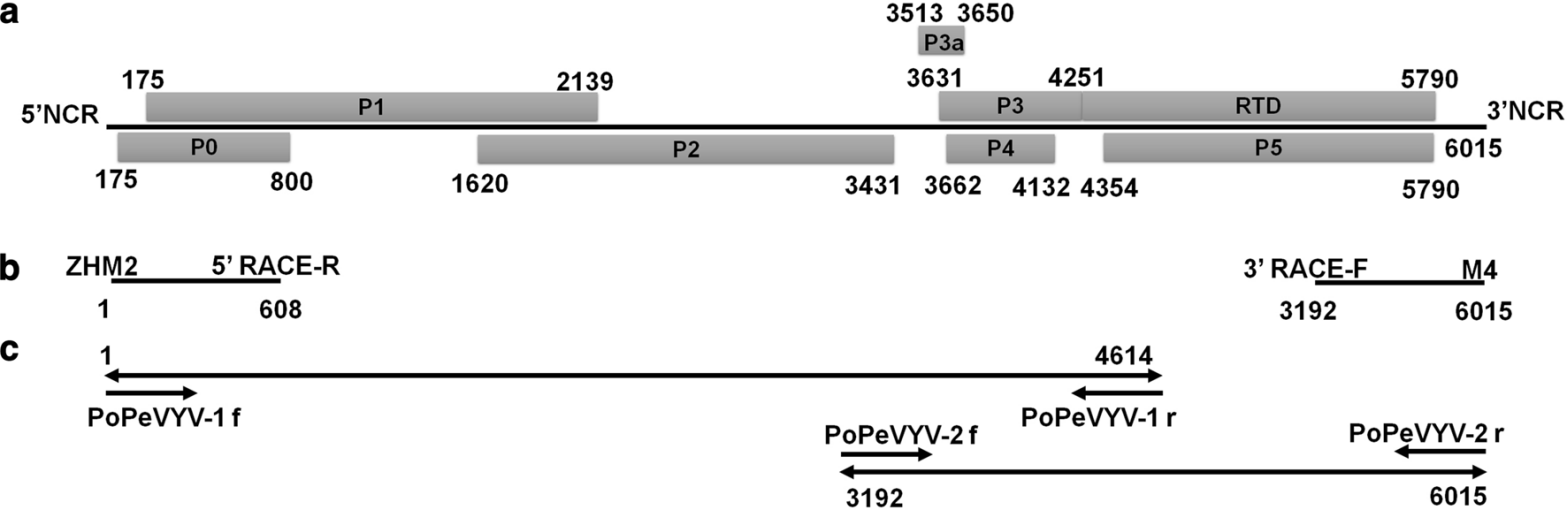
**a** Genome organization PoPeVYV. P1-P2 fusion protein is expressed as a fusion protein with P1 through a translational -1 frameshift, and encodes the RdRp (nts 175–1620, 1620–3431); P3a is expressed by initiation at an AUA codon located at position 3513; P3-P5 protein is translated via a readthrough of the termination codon at the end of P3 (nts 3631–4249, 4252–5790). **b**, **c** Diagram showing how the full-length sequence was constructed by overlap extension RT-PCR

In order to acquire the full-length sequence of PoPeVYV in pod pepper, 5′ and 3′ RACE reactions were then performed to obtain the complete terminal sequences. In brief, the 3′ end of the viral RNA was polyadenylated using Poly(A) Tailing Kit (Invitrogen) and first-strand cDNA was synthesized using M4T primer (Additional file 1: Table S1). The 3′ terminal end was PCR amplified from the cDNA using M4 and 3′-RACE-F (Additional file 1: Table S1). Similarly, the 5′ end of the cDNA, after purification by treatment with Gel extraction Kit (Omega) was polyadenylated using the Poly(A) Tailing Kit. The purified cDNA/RNA heterocomplex was ligated with ZHM1 using T4 RNA ligase (Thermo Scientific) and PCR amplified using ZHM2 and 5′ RACE-R (Additional file 1: Table S1; Fig. 1a, b). To avoid errors in sequence assembly, the whole viral sequence was then amplified using two overlapping sections with the primer pairs PoPeVYV-1 and PoPeVYV-2 (Additional file 1: Table S1; Fig. 1c). RT-PCR products (expected sizes 4614 bp and 2824 bp) were obtained using the methods of KOD-plus-Neo (Toyobo) and the two products (which overlapped by 1423 bp) were cloned into the pGEM-T Easy Vector (Promega) and sequenced. We obtained 14 amplicons of PoPeVYV-1 and 4 amplicons of PoPeVYV-2, and found that 12 amplicons of PoPeVYV-1 exactly matched the overlapping region of PoPeVYV-2. The complete sequence was 6015 nt long (GenBank accession number: MT188667).

PoPeVYV has a genome organization characteristic of members of the genus *Polerovirus*, with seven predicted genes encoding proteins P0 to P5 and P3a [6] (Fig. 1a). Over its entire genome our new virus is related (87.8% nucleotide identity) to Tobacco vein distorting virus (TVDV, accession EF529624) and had 85.0% nt identity to the (Chinese) PeVYV-3 (accession KP300822) (Table 1). However, the 5′ half (nts 1–4251) of PoPeVYV has 93.1% nt identity to the corresponding region of PeVYV-3, and 87.7% nt identity to that of TVDV. The 3′ half (nts 4252–6015) of PoPeVYV has only 64.1% nt identity with PeVYV-3, but 91.7% nt identity with TVDV (Additional file 3: Fig. S2). These results indicated that PoPeVYV in pod pepper might be a recombined virus with PeVYV-3 and TVDV as parents.

**Table 1:** Identity percentages between the genomes of PoPeVYV and with those of closely related species

	5′NCR	P0		P1		P2		iNCR	P3a		P3		P4		RTD		3′NCR	Full genome
	nt	aa	nt	aa	nt	aa	nt	nt	aa	nt	aa	nt	aa	nt	aa	nt	nt	nt
PoPeVYV	100	100	100	100	100	100	100	100	100	100	100	100	100	100	100	100	100	100
PeVYV-1	**98.0**	83.5	88.9	89.8	91.9	93.4	93.3	**96.3**	97.8	97.8	94.2	93.1	89.7	94.7	49.5	55.8	92.4	84.6
PeVYV-2	**98.0**	83.9	88.9	89.9	92.1	94.7	93.8	92.5	91.1	92.0	93.1	92.8	87.6	93.3	65.9	68.9	88.0	87.0
PeVYV-3	**98.0**	**87.1**	**90.5**	**90.8**	**92.6**	93.9	92.7	95.1	**100**	**97.8**	**94.2**	**94.7**	90.4	94.7	49.7	60.6	91.6	85.0
PeVYV-4	66.0	85.1	89.2	90.1	91.5	94.4	93.7	92.5	95.6	94.9	92.7	92.6	91.0	94.5	49.1	55.8	92.4	84.1
PeVYV-5	96.0	77.5	85.2	87.2	90.3	95.0	93.5	92.5	93.3	94.9	94.2	94.4	**92.9**	**95.8**	51.8	57.2	**92.7**	84.1
PeVYV-6	96.0	85.1	89.6	90.5	92.9	**95.0**	**94.1**	90.0	95.6	94.2	94.7	94.2	92.3	95.8	51.8	58.4	**92.7**	85.3
TVDV	81.6	76.3	81.3	77.2	83.0	90.8	88.6	84.8	95.6	87.7	89.8	92.2	84.6	90.9	**89.7**	**91.9**	88.6	**87.8**

^a^*NCR* noncoding region, *iNCR* intergenic NCR, *aa* amino acid, *nt* nucleotide. Highest percentages are underlined and in bold

^b^PeVYV-1 (*Pepper vein yellows virus-1*, AB594828), PeVYV-2 *(Pepper vein yellows virus-2*, HM439608), PeVYV-3 (*Pepper vein yellows virus-3*, KP300822), PeVYV-4 (*Pepper vein yellows virus-4*, KU999109), PeVYV-5 (*Pepper vein yellows virus-5*, KY523072), PeVYV-6 (*Pepper vein yellows virus-6*, LT559483), TVDV (*Tobacco vein distorting virus*, EF529624), PoPeVYV (Pod pepper vein yellows virus, MT188667)

**Fig. 2:**
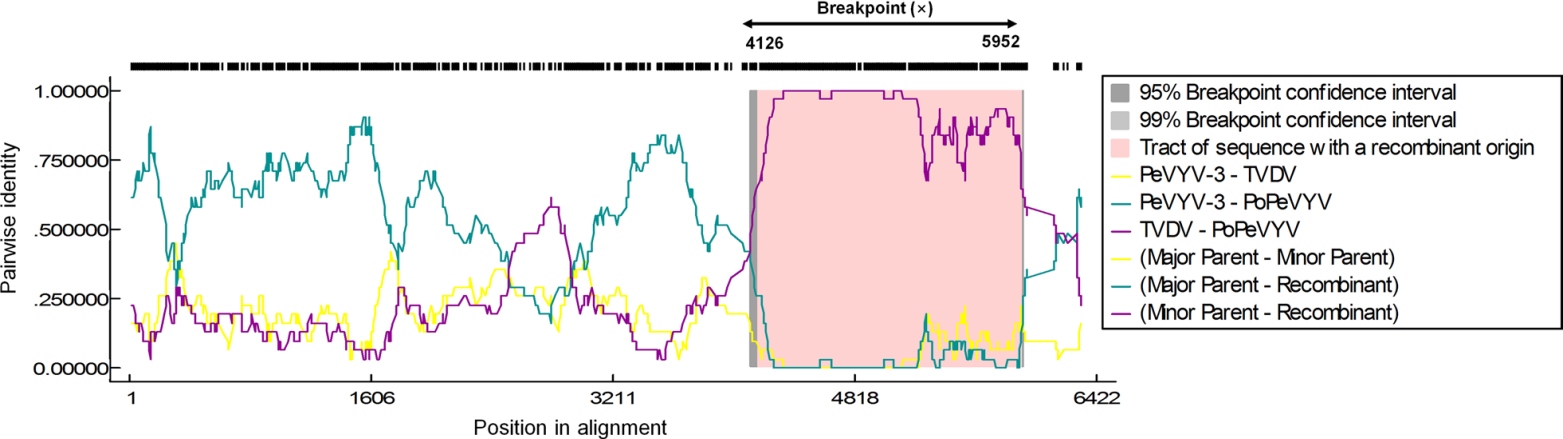
Recombination (RDP) and genetic map of PoPeVYV showing major (PeVYV-3) and minor (TVDV) parents and the transition in the genome sequence at the breakpoint (X; 4126–5952 bp); the recombinant region is highlighted in pink. Multiple nucleotide sequences were aligned using MUSCLE, and then analyzed using RDP4 with default parameters

**Fig. 3:**
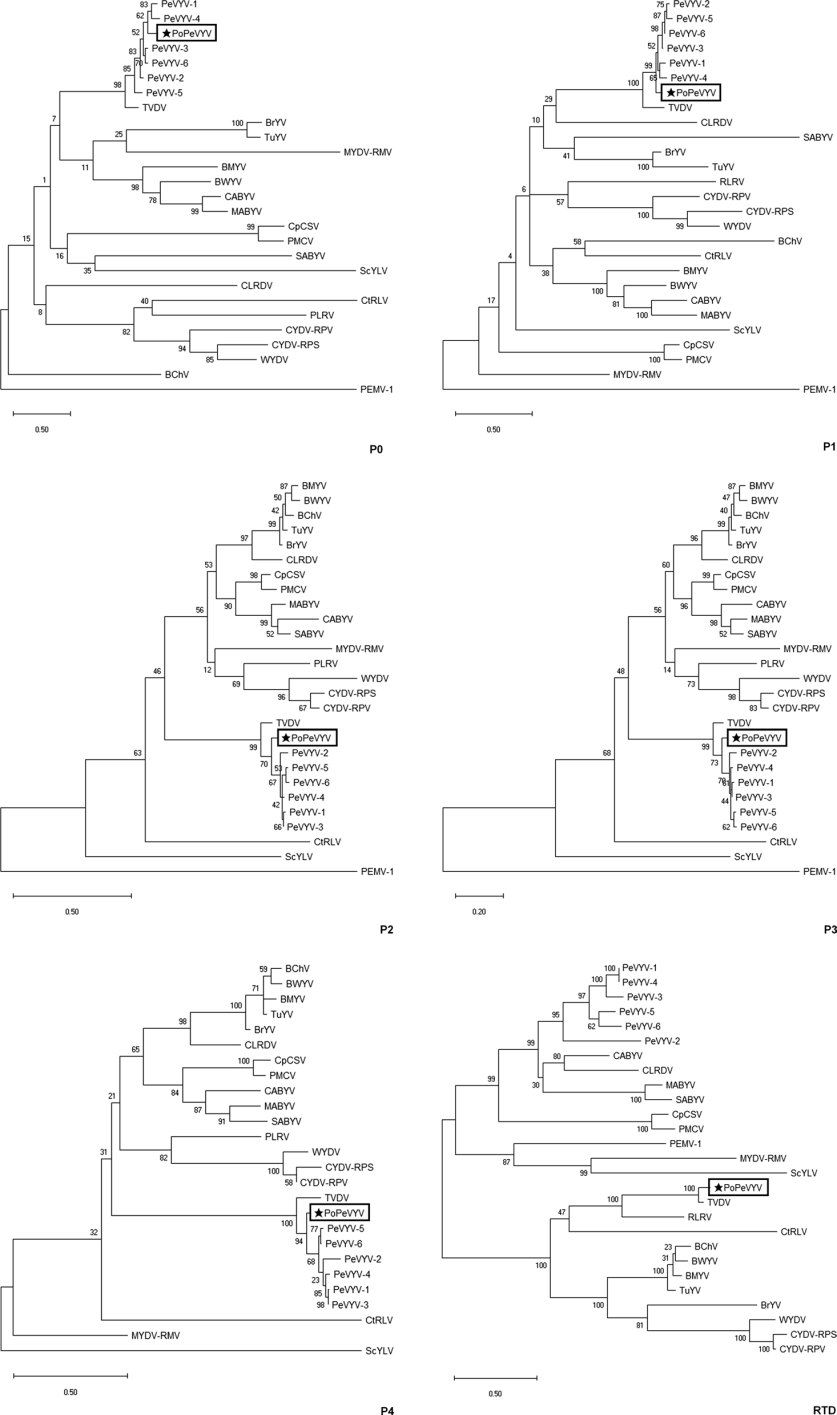
Maximum Likelihood phylogenetic trees constructed using MEGA X, showing the relationship of PoPeVYV to other members of the family *Luteoviridae* using amino acid sequences of the different gene products. Except for P4, the enamovirus *Pea enation mosaic virus 1* (PEMV-1, NC_003629.1) was used as an outgroup. Numbers on branches are bootstrap support values (1000 replicates). The models employed were LG + G for P0, JTT + G for P1, LG + G + F for P2, JTT + G for P3, JTT + G + I for P4 and WAG + G for RTD, respectively. The virus abbreviations and accession numbers are as follows: BChV (*Beet chlorosis virus*, AF352024), BMYV (*Beet mild yellowing virus*, DQ132996), BrYV (*Brassica yellows virus*, KF015269), BWYV (*Beet western yellows virus*, AF473561), CABYV (*Cucurbit aphid-bome yellows virus*, JQ700305), CLRDV (*Cotton leafroll dwarf virus*, NC_014545), CpCSV (*Chickpea chlorotic stunt virus*, AY956384), CtRLV (*Carrot red leaf virus*, AY695933), CYDV-RPS (*Cereal yellow dwarf virus-RPS*, AF235168), CYDV-RPV (*Cereal yellow dwarf virus-RPV*, EF521927), MABYV (*Melon aphid-borne yellows*, JQ700307), MYDV-RMV (*Maize yellow dwarf virus-RMV*, NC_021484), PeVYV-1 (*Pepper vein yellows virus-1*, AB594828), PeVYV-2 *(Pepper vein yellows virus-2*, HM439608), PeVYV-3 (*Pepper vein yellows virus-3*, KP300822), PeVYV-4 (*Pepper vein yellows virus-4*, KU999109), PeVYV-5 (*Pepper vein yellows virus-5*, KY523072), PeVYV-6 (*Pepper vein yellows virus-6*, LT559483), PLRV (*Potato leafroll virus*, AF453390), PMCV(*Pea mild chlorosis virus*, JF507725), SABYV (*Suakwa aphid-bome yellows virus*, JQ700308), ScYLV(*Sugarcane yellow leaf virus*, JF925154), TuYV (*Tumip yellows virus*, X13063), TVDV (*Tobacco vein distorting virus*,EF529624), and WYDV (*Wheat yellow dwarf virus*, FM865413)

Poleroviruses are prone to recombination among themselves or with viruses belonging to other genera and the relationships between PoPeVYV, the previously described PeVYV isolates and TVDV suggests that there has been a recombination event affecting the 3′-end of the genome. This was confirmed using a variety of methods on the RDP4 recombinant platform [11]. A single recombination event affecting nucleotide positions 4126–5952, with TVDV and PeVYV-3 as the respective minor and major parents was consistently identified using GENECONV (P value of 1.035 E-93), RDP (8.410 E-85), BootScan (7.726 E-80), MaxChi (4.294 E-35), Chimera (7.719 E-05), SiScan (3.341 E-68), and Phylpro (2.331 E−15) (Fig. 2). Alignment of amino acid sequences of the PoPeVYV proteins with those of the six species of PeVYVs and TVDV also indicated a recombination event (Table 1). Phylogenetic trees were constructed from the complete genome sequences, 5′- half or 3′- half of other poleroviruses and an enamovirus as an outgroup (Additional file 4: Fig. S3). PoPeVYV, PeVYVs and TVDV formed an independent group separate from the other poleroviruses. The analysis confirmed that the genome and 3′ half of PoPeVYV was similar to TVDV, but that the 5′ half of PoPeVYV was similar to the PeVYVs (Additional file 4: Fig. S3). This is reflected in the phylogenetic analysis of the amino acid sequences of the separate gene products [12]: the P0 of PoPeVYV is most similar to PeVYV-1/4, P1/P2/P3/P4 are closely related to other PeVYVs but the RTD is most similar to TVDV (Fig. 3). However, proteins encoded by the 5′ half of the genome (P0 to P4) had the highest identity to those of PeVYV-3 and PeVYV-6, whereas proteins translated from the 3′ half (P5/RTD) were more closely related to those of TVDV (Table 1, Fig. 3). The various PeVYVs recognized usually have distinct P0 protein sequences and the topology of the P0 phylogenetic tree may depict the ongoing selection for a protein exhibiting better RNA silencing suppression capacity after a recent host jump [4, 7, 13]. The P0 of PoPeVYV has 77.5–87.1% amino acid identity and 85.2–90.5% nucleotide identity to that of the other PeVYVs, and 76.3% amino acid identity (81.3% nucleotide identity) to that of TVDV (Table 1). In the P0 phylogenetic tree PoPeVYV was most similar to PeVYV-1 and PeVYV-4 and was clearly more similar to the PeVYVs than to TVDV (Fig. 3). These results suggest that PoPeVYV is a new recombinant polerovirus.

**Fig. 4:**
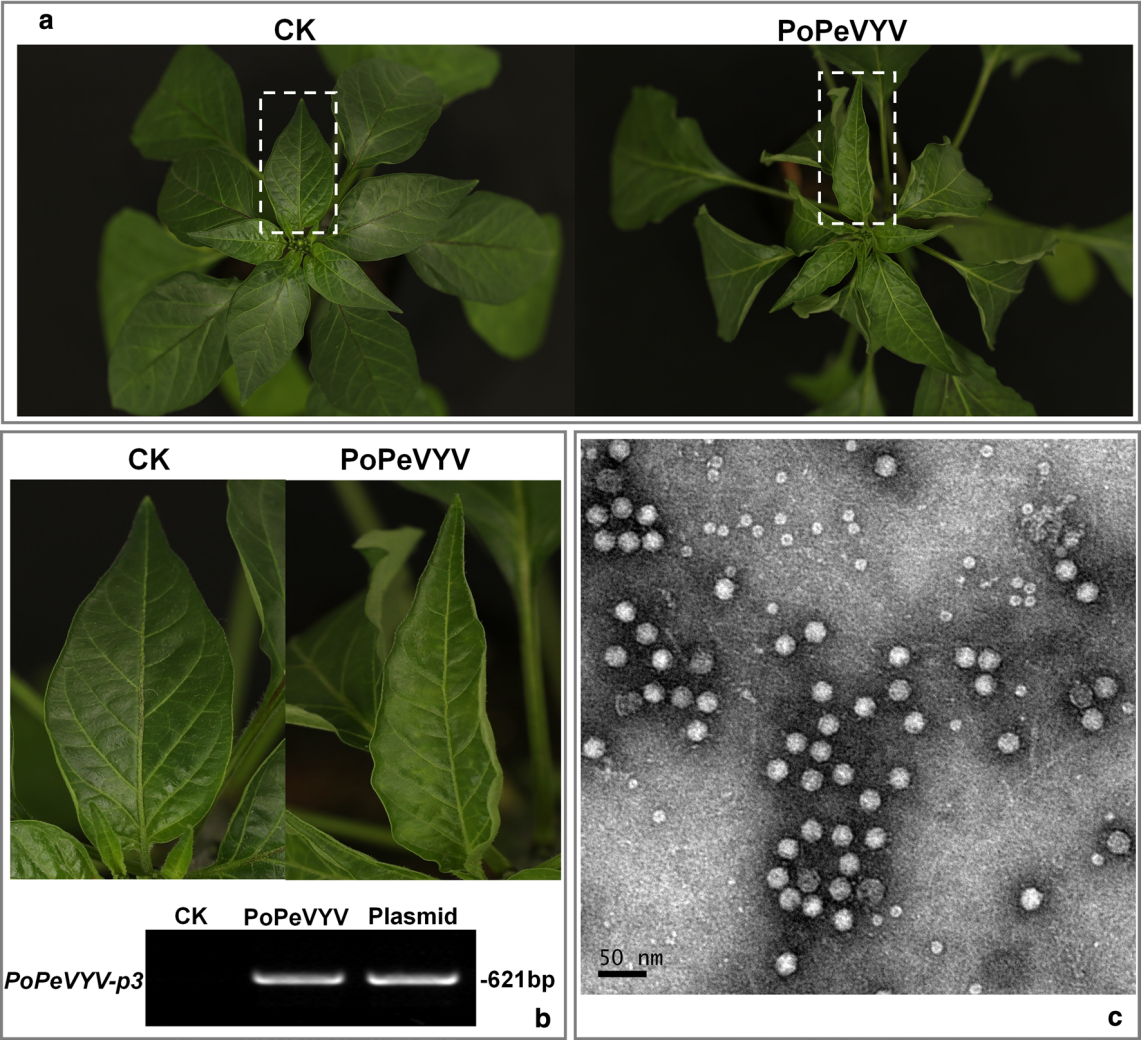
Symptoms caused by PoPeVYV infectious clone in *C. frutescens*. **a** Phenotype of *C. frutescens* plants agroinfiltrated with PoPeVYV infectious clone or empty agrobacterium (CK) 45 days post infiltration. **b** RT-PCR confirming the presence of viral RNA in systemic leaves of inoculated plants. A 621-bp fragment of the p3 was amplified, with a plasmid containing the p3 as a positive control. **c** Virions purified from leaves infected by the PoPeVYV infectious clone and observed by TEM

An infectious clone of the virus was constructed for further investigation. RT-PCR was performed using KOD-plus-Neo (Toyobo) following the manufacturer’s protocol. To generate infectious clones, the CloneExpress MultiS One Step Cloning Kit (Vazyme) was used for homologous recombination. Two overlapped PCR products were amplified with primers (Inf-PoPeVYV-1/2), and recombined with the linearized binary vector pCB301-MD (a modified version of pCB301 [14]), which includes the double 35S promoter and nopaline synthase terminator (NOS) (Additional file 5: Fig. S4) [15, 16]. In this way, the full-length PoPeVYV cDNA was inserted between an upstream 35S promoter and a downstream hepatitis delta virus (HDV) ribozyme and NOS terminator in the binary vector to construct pCB-PoPeVYV. The PoPeVYV cDNA was ligated with 3′-terminal of 35S promoter without any extra nucleotide (Additional file 6: Fig. S5) [15, 16]. This clone was transformed into *Agrobacterium tumefaciens* which was then delivered to *C. frutescens* plantlets by infiltration. There was mild upward leaf curling 45 days after inoculation (Fig. 4a), and RT-PCR using primers to detect the coat protein gene in the newly-emerged non-inoculated leaves showed that viral RNA was present and had spread systemically in all the inoculated plants (12/12) but not in the controls (Fig. 4b). Virions were purified from *C. frutescens* leaves using the method described previously [17]. Isometric particles about 25 nm in diameter were observed in the purified preparation from the inoculated plants (Fig. 4c) but not from the controls. RT-PCR and subsequent sequencing confirmed that the virus in the symptomatic (systemic) leaves had the same sequence as the PoPeVYV cDNA clone inoculated (data not show). These results demonstrate the infectivity of the full-length PoPeVYV to *C. frutescens*.

In fields of cultivated pod pepper in Wenshan, we had observed severe yellowing and curling symptoms with small leaves (Additional file 2: Fig. S1). Those symptoms were more severe than we observed in plants inoculated with the infectious clone of PoPeVYV by *Agrobacterium tumefaciens* which also did not have the obvious vein yellowing usually caused by PeVYVs. Previous studies have shown that the P0 proteins of poleroviruses display variable RNA silencing suppression activity, and induce distinct symptoms [18, 19]. The P0 of PoPeVYV has only 77.5–87.1% amino acid identity to the other PeVYVs, which could account for the distinct symptoms but the field plants were also infected with other viruses (PeVYVs, ChiVMV, ChiRSV, CMV etc.) as frequently happens in the field and which has also been reported in Taiwan and Thailand [20, 21]. The severe viral symptoms in the field may therefore be a synergistic effect of mixed infection.

Recombination is an important source of genetic variability in viruses, particularly for viruses possessing an RNA genome. PeVYVs have higher identities to TVDV in the 5′ half of the genome and are considered to be recombinants of TVDV and other poleroviruses [4, 7, 8]. The new recombinant identified here has higher identity to TVDV at the 3′ part of genome, indicating a different sort of recombinant event. The 5′ half of the PoPeVYV genome shares high identity with PeVYV-3, which was reported from Hunan province in China [3]. The 3′ half is much more distant from PeVYV-3 but is more homologous with that of TVDV. TVDV cause a devastating tobacco disease in many tobacco producing areas include Hunan, Guizhou and Yunnan provinces in China [22, 23]. Additionally, PeYVY has been reported to infect tobacco in Guizhou province suggesting that co-infection of tobacco might have provided an opportunity for recombination [24]. Recombination poses a problem for classification. The currently-recommended species demarcation criteria in the family *Luteoviridae* suggest that different species should have > 10% difference in amino acid sequence identity in any gene product from their closest relative. The P0 and P5 proteins of PoPeVYV have respectively 12.9–23.7% and 10.3–70.5% difference in amino acid identity to those of PeVYV1-6 (Table 1). If applied here, those criteria suggest that PoPeVYV could be representative of a distinct species.

## Conclusions

A full-length sequence of Pod pepper vein yellows virus (MT188667) was determined. Alignment and recombination analysis predicted a single recombination event with PeVYV-3 as the major parent but with the region 4126–5192 nt derived from TVDV as the minor parent. PoPeVYV is a new recombinant polerovirus infecting *C. frutescens* in Yunnan province, China.

## Supplementary Information

**Additional file 1.** Primers used in this study. **a**. The Sequence validation primers were used to verify the virus sequences. **b**. The RACE PCR primers were used to obtain the complete terminal sequences of PoPeVYV. **c**. The PCR detection primers were used to detect the virus. **d**. The Colony PCR and Linearize vector primers were used to generate infectious clones (pCB-PoPeVYV).

**Additional file 2.** Symptoms of virus-infected Pod peppers from the field.

**Additional file 3.** Pairwise nucleotide sequence comparisons of PoPeVYV with related reference viruses. Genome: the full genome sequence of viruses; 5’- half: sequence contain the 5’ NCR to P3; 3’- half: sequence from P3 readthrough domain to 3’ NCR. Multiple nucleotide sequences were aligned using MUSCLE, and pairwise nucleotide sequence comparisons were done using the SDT (Species Demarcation Tool) v1.2 program.

**Additional file 4.** Phylogenetic tree of PoPeVYV. The enamovirus Pea enation mosaic virus 1 (PEMV-1, NC_003629.1) was used as an outgroup. The evolutionary history was inferred using the Neighbor-Joining method. The percentage of replicate trees in which the associated taxa clustered together in the bootstrap test (1000 replicates) are shown next to the branches. The evolutionary distances were computed using the Maximum Composite Likelihood method and are in the units of the number of base substitutions per site. Evolutionary analyses were conducted in MEGA X.

**Additional file 5.** Structure of the cloning vector pCB301-MD. The 2x35S in pCB301-MD was originally from the vector pCass2 [15], MCS- HDVRZ-NOS cassette was originally from the vector pHST40 [16]. Transcription Start site and Ribozyme cleavage site were based on vectors of pCass2 and pHST40 in references.

**Additional file 6.** The strategy for constructing the full-length infectious clone by homologous recombination. Two overlapped PCR products of PoPeVYV were amplified with primers (Inf-PoPeVYV-1 f/r, Inf-PoPeVYV-2 f/r) and recombined with the linearized binary vector pCB301-MD which was amplified with primers pair Vec-pCB301 (Vec-pCB301 f/r).

**Additional file 7.** The dataset used for the recombinant analysis.

**Additional file 8.** NCBI-generated GenBank file showing the PoPeVYV sequence.

## Data Availability

The complete genome sequences of PoPeVYV were submitted to the GenBank (http://www.ncbi.nlm.nih.gov/genbank/) and the accession number is MT188667.

## Abbreviations

nt: Nucleotide
RACE: Rapid amplification of cDNA ends
RT-PCR: Reverse transcription polymerase chain reaction
PoPeVYV: Pod Pepper vein yellows virus
PeVYV: Pepper vein yellows virus
TVDV: Tobacco vein distorting virus
