# Supplementary material for: Pod pepper vein yellows virus, a new recombinant polerovirus infecting Capsicum frutescens in Yunnan province, China
Source: Virol J. 2021 Feb 23;18:42. doi: 10.1186/s12985-021-01511-5 (PMC7901092; doi:10.1186/s12985-021-01511-5)
Supplement: Supplementary file 1 — Additional file 1. Primers used in this study. a. The Sequence validation primers were used to verify the virus sequences. b. The RACE PCR primers were used to obtain the complete terminal sequences of PoPeVYV. c. The PCR detection primers were used to detect the virus. d. The Colony PCR and Linearize vector primers were used to generate infectious clones (pCB-PoPeVYV). [file 12985_2021_1511_MOESM1_ESM.docx]

Table S1. Primers used in this study

| Objective | name | sequence |
| --- | --- | --- |
| Sequence validation | PoPeVYV-1 f | 5′-ACAAAATATACGAAGAGAGAGAG -3′ |
|  | PoPeVYV-1 r | 5′-TAACCCATCACTCCTCCCAC-3′ |
|  | PoPeVYV-2 f | 5′-GGACAACTGGAATTCTGCTC-3′ |
|  | PoPeVYV-2 r | 5′-ACATCATAGACCAGGGGGGGGTATCTATAC -3′ |
| RACE PCR | M4 | 5′-GTTTTCCCAGTCACGAC-3′ |
|  | M4T | 5'-GTTTTCCCAGTCACGAC(T)_15_-3′ |
|  | ZHM2 | 5'-gAggAgggAggggAAgAg-3′ |
|  | ZHM1 | 5'-PO_4_-CTCTTCCCCTCCCTCCTC-NH_2_-3′ |
|  | 3' -RACE-F | 5'-CCCGTCAACAAAGACTACATTC-3′ |
|  | 5'-RACE-R | 5'-GGAGACAATGACCCAGATG-3′ |
| PCR detection | PeVYV-CP f | 5′-ATGAATACGGGAGGAGTTAGG -3′ |
|  | PeVYV-CP r | 5′-CTATTTGGGGTTGTGCAGTTG -3′ |
|  | Ca-actin f | 5′-CAGCCTCTTGTCTGTGATAATG-3′ |
|  | Ca-actin r | 5′-GAGCATAACCTTCATAGATGGG-3′ |
| Colony PCR | Inf-PoPeVYV-1 f | 5′-AAGTTCATTTCATTTGGAGAGGACAAAATATACGAAGAGAGAGAG-3′ |
|  | Inf-PoPeVYV-1 r | 5′-GAGCAGAATTCCAGTTGTCC-3′ |
|  | Inf-PoPeVYV-2 f | 5′-GGACAACTGGAATTCTGCTC-3′ |
|  | Inf-PoPeVYV-2 r | 5′-TGGAGATGCCATGCCGACCCACATCATAGACCAGGGGGGGGTATCTATACACCCCCCTACCCTAAGAATT-3′ |
| Linearize vector | Vec-pCB301 f | 5'-GGGTCGGCATGGCATCTCCA-3′ |
|  | Vec-pCB301 r | 5'-CCTCTCCAAATGAAATGAACTT-3′ |

1. The Sequence validation primers were used to verify the virus sequences.
2. The RACE PCR primers were used to obtain the complete terminal sequences of PoPeVYV.
3. The PCR detection primers were used to detect the virus.
4. The Colony PCR and Linearize vector primers were used to generate infectious clones (pCB-PoPeVYV).
