# Supplementary figures and images for: Pod pepper vein yellows virus, a new recombinant polerovirus infecting Capsicum frutescens in Yunnan province, China
Source: Virol J. 2021 Feb 23;18:42. doi: 10.1186/s12985-021-01511-5 (PMC7901092; doi:10.1186/s12985-021-01511-5)

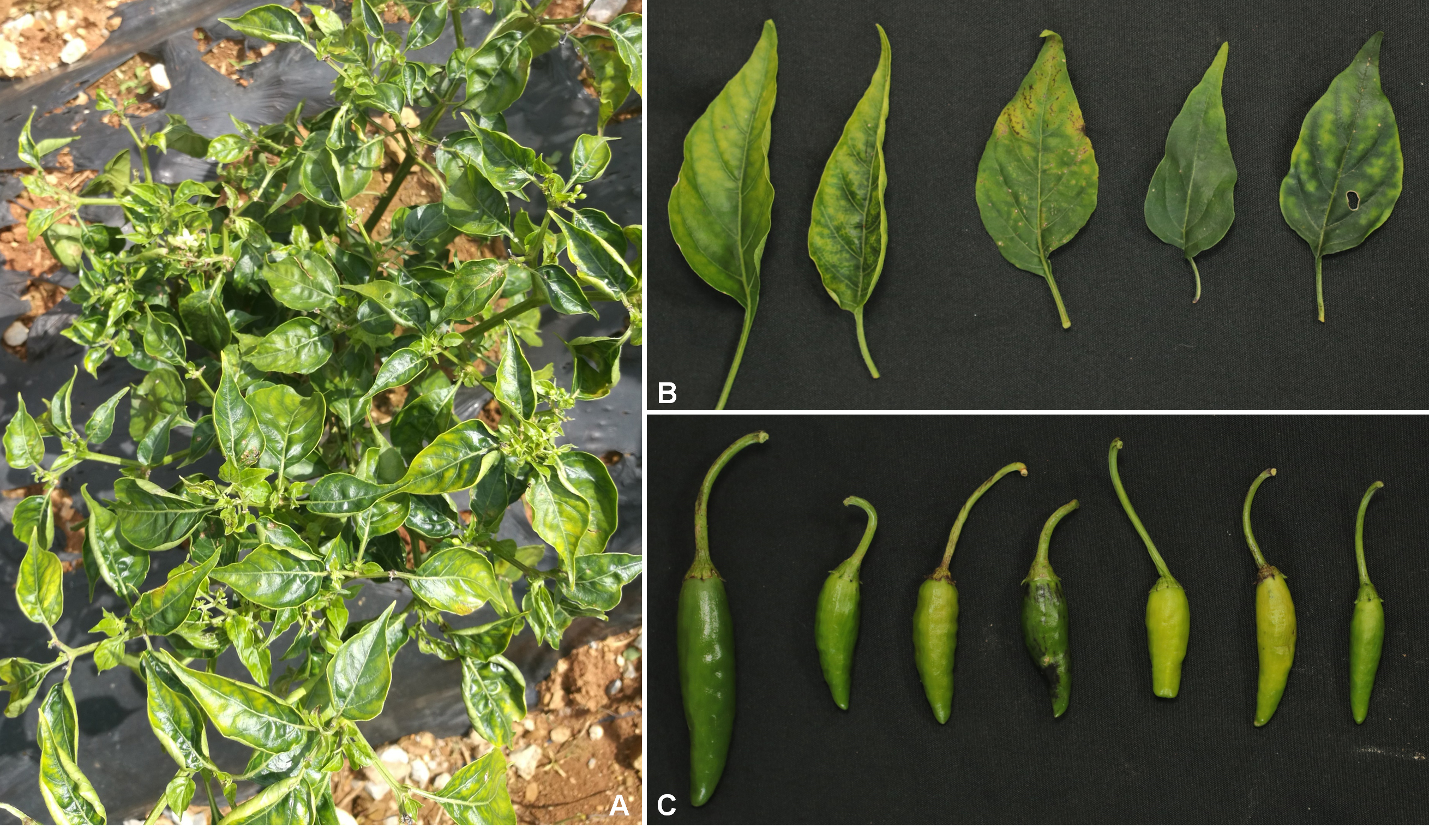

Supplement: Supplementary file 2 — Additional file 2. Symptoms of virus-infected Pod peppers from the field. [file 12985_2021_1511_MOESM2_ESM.jpg]

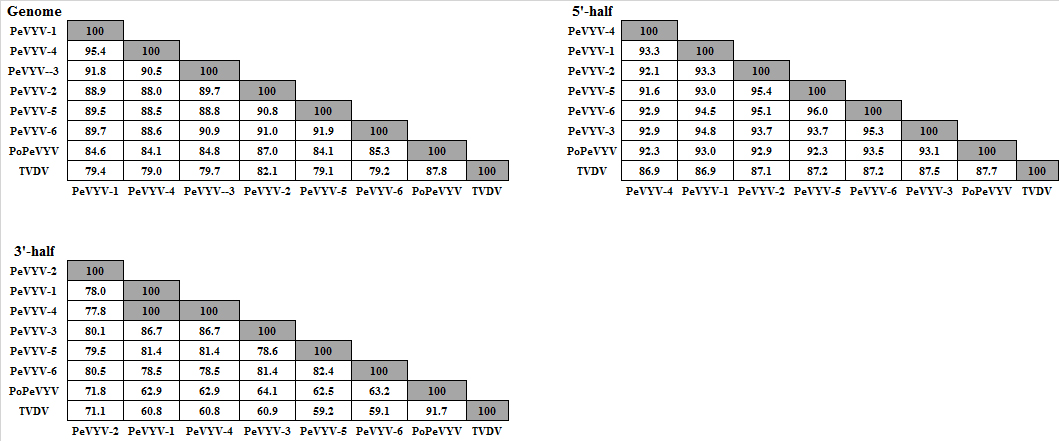

Supplement: Supplementary file 3 — Additional file 3. Pairwise nucleotide sequence comparisons of PoPeVYV with related reference viruses. Genome: the full genome sequence of viruses; 5’- half: sequence contain the 5’ NCR to P3; 3’- half: sequence from P3 readthrough domain to 3’ NCR. Multiple nucleotide sequences were aligned using MUSCLE, and pairwise nucleotide sequence comparisons were done using the SDT (Species Demarcation Tool) v1.2 program. [file 12985_2021_1511_MOESM3_ESM.jpg]

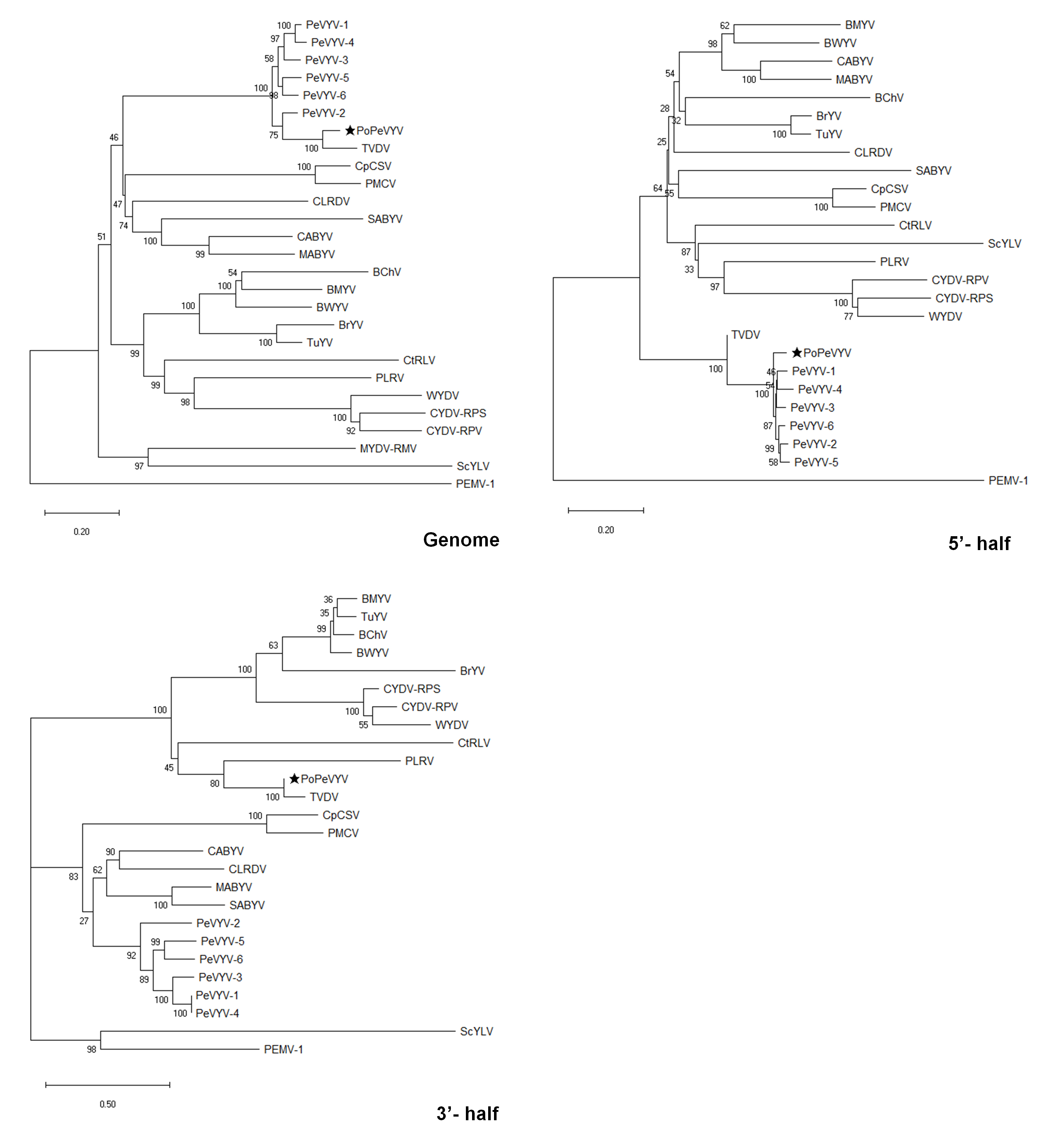

Supplement: Supplementary file 4 — Additional file 4. Phylogenetic tree of PoPeVYV. The enamovirus Pea enation mosaic virus 1 (PEMV-1, NC_003629.1) was used as an outgroup. The evolutionary history was inferred using the Neighbor-Joining method. The percentage of replicate trees in which the associated taxa clustered together in the bootstrap test (1000 replicates) are shown next to the branches. The evolutionary distances were computed using the Maximum Composite Likelihood method and are in the units of the number of base substitutions per site. Evolutionary analyses were conducted in MEGA X. [file 12985_2021_1511_MOESM4_ESM.jpg]
